# Long-term vemurafenib treatment drives inhibitor resistance through a spontaneous KRAS G12D mutation in a BRAF V600E papillary thyroid carcinoma model

**DOI:** 10.18632/oncotarget.9023

**Published:** 2016-04-26

**Authors:** Brian P. Danysh, Erin Y. Rieger, Deepankar K. Sinha, Caitlin V. Evers, Gilbert J. Cote, Maria E. Cabanillas, Marie-Claude Hofmann

**Affiliations:** ^1^ Department of Endocrine Neoplasia and Hormonal Disorders, The University of Texas MD Anderson Cancer Center, Houston, TX, USA; ^2^ Diagnostic Genetics Program, School of Health Professions, The University of Texas MD Anderson Cancer Center, Houston, TX, USA

**Keywords:** BRAF V600E, kinase inhibitor, resistance, KRAS G12D, papillary thyroid cancer (PTC)

## Abstract

The BRAF V600E mutation is commonly observed in papillary thyroid cancer (PTC) and predominantly activates the MAPK pathway. Presence of BRAF V600E predicts increasing risk of recurrence and higher mortality rate, and treatment options for such patients are limited. Vemurafenib, a BRAF V600E inhibitor, is initially effective, but cells inevitably develop alternative mechanisms of pathway activation. Mechanisms of primary resistance have been described in short-term cultures of PTC cells; however, mechanisms of acquired resistance have not. In the present study, we investigated possible adaptive mechanisms of BRAF V600E inhibitor resistance in KTC1 thyroid cancer cells following long-term vemurafenib exposure. We found that a subpopulation of KTC1 cells acquired resistance to vemurafenib following 5 months of treatment with the inhibitor. Resistance coincided with the spontaneous acquisition of a KRAS G12D activating mutation. Increases in activated AKT, ERK1/2, and EGFR were observed in these cells. In addition, the resistant cells were less sensitive to combinations of vemurafenib and MEK1 inhibitor or AKT inhibitor. These results support the KRAS G12D mutation as a genetic mechanism of spontaneously acquired secondary BRAF inhibitor resistance in BRAF V600E thyroid cancer cells.

## INTRODUCTION

Although the majority of patients with papillary thyroid cancer (PTC) have excellent long-term outcomes with standard therapy, up to approximately 25% have a more aggressive course, presenting with or developing distant metastases. The BRAF V600E mutation is an established oncogenic driver that is observed at high frequency in melanoma and PTC [1–4]. Its presence strongly correlates with aggressive tumor characteristics, such as metastasis and disease recurrence [2, 5], radioactive iodine resistance, and patient mortality [6, 7]. The mutation constitutively activates the catalytic activity of the BRAF kinase, a member of the RAF family of serine/threonine enzymes. This activity leads to activation of MEK and ERK1/2 kinases and results in functional dependence of the cells upon the BRAF/MEK/ERK cascade for growth and survival.

Inhibitors of BRAF V600E are being evaluated in the clinic for patients with BRAF-mutated PTC. The selective BRAF V600E inhibitors vemurafenib and dabrafenib have shown promise in clinical trials [8–10]. Although these results are quite favorable, as with all kinase inhibitors, resistance ultimately develops. Acquired resistance to BRAF V600E mutation inhibition presents a significant therapeutic challenge in thyroid cancer patients. Identifying and better understanding the mechanisms that render mutant BRAF-expressing cells resistant to this therapy is critical to improving patient outcomes. Research has thus recently focused on elucidating possible mechanisms of resistance to BRAF V600E inhibitors.

Multiple mechanisms have been proposed for how the tumors escape the inhibitory control. Described intrinsic (RAF/MEK/ERK pathway) and extrinsic (alternative pathways) mechanisms of acquired BRAF inhibitor resistance in thyroid cancer include: RAF/MEK/ERK pathway activation brought about through alternate BRAF splicing [11], activation of the PI3K/AKT pathway through c-MET [12], autocrine NRG1- mediated HER3 receptor activation of the PI3K/AKT and RAS/RAF/MEK/ERK pathways [13], and autocrine IL-6-mediated JAK/STAT3 and RAS/RAF/MEK/ERK pathway activation [14]. Recently, Duquette and colleagues described the genomic co-occurrence of the BRAF V600E mutation with either *MCL1* (pro-survival factor) copy number gain or *P16* (tumor suppressor) loss. They demonstrated the association of these genomic alterations with metastatic PTC and primary resistance to vemurafenib [15]. In addition to activation of intrinsic and extrinsic signaling pathways through various mechanisms, genomic heterogeneity of cancer cells under drug selection may accelerate clonal evolution and emergence of more aggressive genotypes, or select for cancer stem-like cells. To investigate possible adaptive mechanisms of BRAF V600E inhibitor resistance, in the present study, we performed long-term exposure experiments of BRAF V600E PTC cells with different doses of the BRAF V600E selective inhibitor vemurafenib and followed the fate of these cells over a time span of 5 months. Our analyses indicated that PTC cells under long-term vemurafenib pressure undergo changes in gene expression associated with thyroid follicular cell dedifferentiation. Further, a subpopulation of PTC cells emerged as heterogeneous for a KRAS G12D mutation, in addition to the existing BRAF V600E mutation, which conferred resistance to BRAF V600E inhibition. This study therefore provides insight into an alternative mechanism of inhibitor resistance through acquisition or selection of hotspot mutations. Understanding PTC tumor heterogeneity and mutational patterns emerging under drug pressure is fundamental to improving clinical studies by identifying alternative drug regimens and will help elucidate mechanisms of disease progression.

## RESULTS

### BCPAP and KTC1 cell lines respond differently to the anti-proliferative effects of vemurafenib

**Figure 1:**
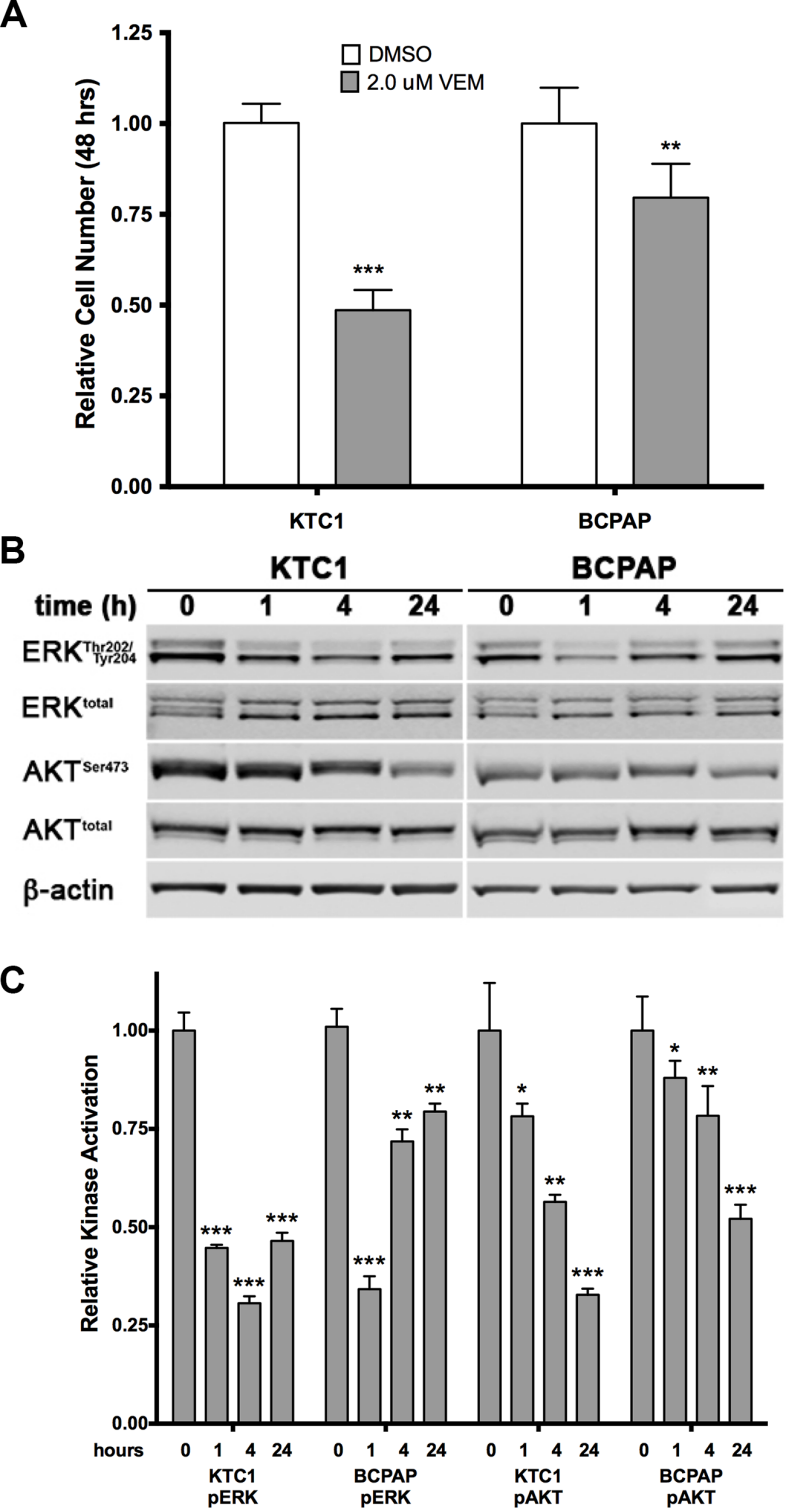
Effects of acute treatment with the BRAF V600E inhibitor vemurafenib on two PTC cell lines (**A**) KTC1 and BCPAP cells were grown for 2 days in presence of 2.0 μM vemurafenib. KTC1 cells are shown to be more sensitive to the BRAF V600E inhibitor than BCPAP cells. (**B**, **C**) Western blot analysis of ERK1/2 and AKT activation in the same cell lines following 1, 4, and 24 hours exposure to 2.0 μM vemurafenib. A sharp reduction in phosphorylated ERK1/2 is maintained in KTC1 cells, but begins to recover in BCPAP cells after 4 hours of treatment. Phosphorylated AKT gradually drops over 24 hours in both lines, but to a lesser degree in the BCPAP line.

The anti-proliferative effects of vemurafenib on the original BCPAP and KTC1 thyroid cancer cell lines were first evaluated in an acute 48-hour growth assay. BCPAP cells are hemizygous and KTC1 cells are heterozygous for BRAF V600E; both contain several other cancer-associated mutations (Supplementary Table 1). As seen in Figure 1A, vemurafenib at a concentration of 2 μM (a clinically achievable blood and tissue concentration [16]) inhibited the growth of KTC1 cells in culture by 51.5%. However, it only decreased BCPAP cell growth by 20.5%. Western blot analysis showed that the anti-proliferative effect of vemurafenib on KTC1 cells was associated with the inhibition of both ERK1/2 and AKT phosphorylation (Figure 1B, 1C), which are downstream of BRAF and PI3K, respectively. However, in BCPAP cells inhibition of ERK1/2 was transient as recovery was observed beginning 4 hours after treatment. It is possible that this recovery from ERK1/2 activation inhibition in BCPAP cells is related to the high affinity of vemurafenib to serum proteins. Salerno and colleagues previously described a decreased activation of ERK1/2 related to serum concentrations in BCPAP cells. However, these experiments were performed using sub-micromolar concentrations of vemurafenib and ultimately had the opposite effects on growth inhibition [17].

### Long-term exposure of KTC1 cells to vemurafenib selects for additional mutations and decreases markers of differentiation

**Figure 2:**
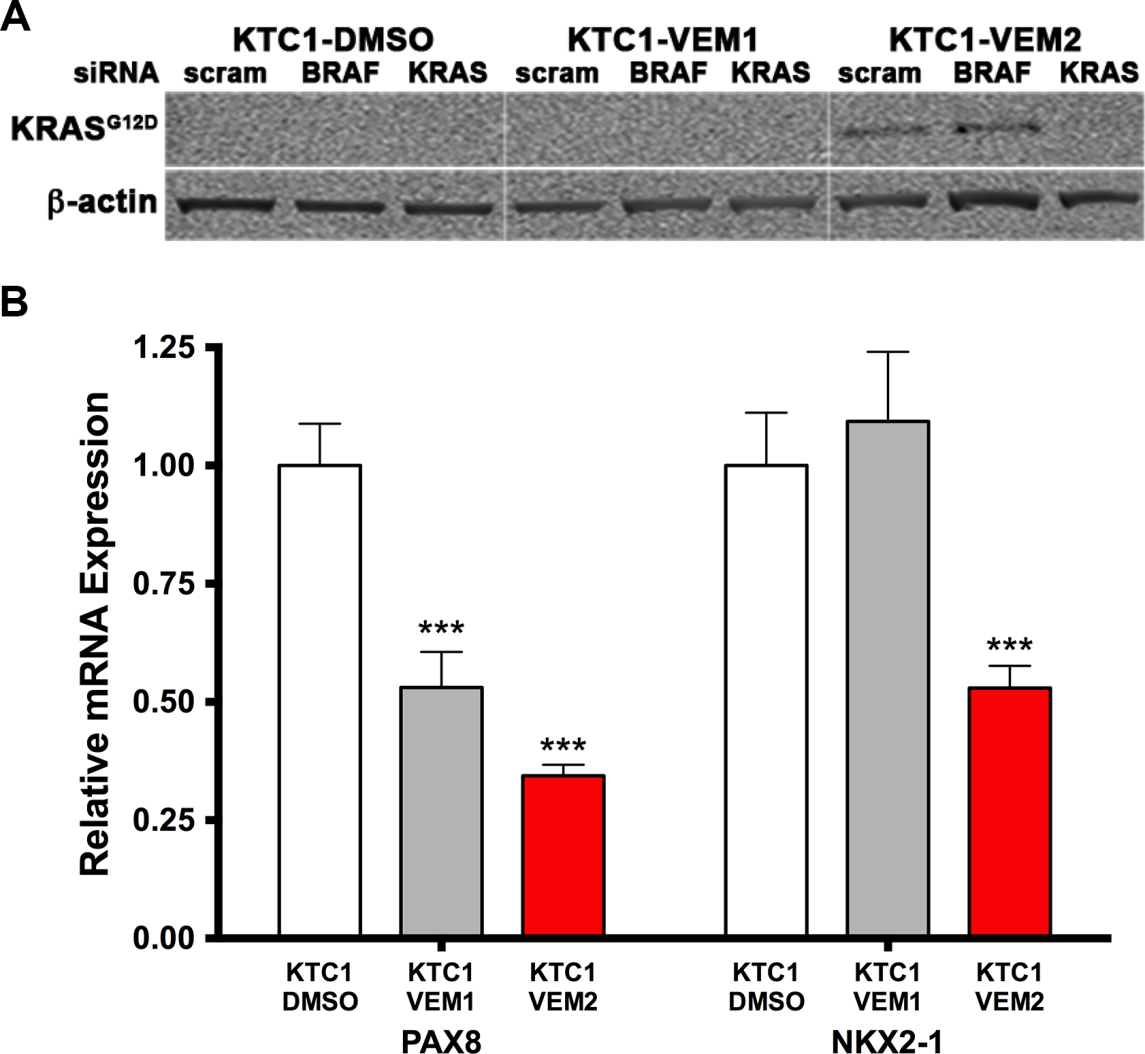
KRAS mutation verification and differentiation marker status in KTC1 subpopulations (**A**) The KRAS G12D mutation in the KTC1-VEM2 cells is verified using a mutation-specific antibody in Western blot analysis of total KRAS and BRAF siRNA knockdowns in the KTC1 subpopulations. Scrambled siRNA was used as a control. (**B**) Quantitative real-time PCR shows the level of dedifferentiation in each subpopulation. Thyroid follicular cell differentiation markers *PAX8* and *NKX2-1* decreased in KTC1-VEM2 (red bars), while only *PAX8* decreased in KTC1-VEM1.

To understand long-term effects of vemurafenib treatment, we continuously exposed KTC1 cells to two different doses of the inhibitor or dimethyl sulfoxide (DMSO) vehicle and followed the fate of these cells over 5 months (20 passages). Three heterogeneous subpopulations of KTC1 cells were obtained and labeled as DMSO (control cells, treated with DMSO vehicle), KTC1-VEM1 (treated with 0.25 μM vemurafenib), and KTC1-VEM2 (treated with 1.0 μM vemurafenib). Short-tandem-repeat (STR) fingerprint analysis (Table 1) indicated that all three subpopulations retained the original KTC1 cell profile published by Schweppe and colleagues [18]. To look for the acquisition of potential “gateway” mutations, we performed a screen for a panel of 420 mutations commonly found in cancers (Supplementary Table 2). The analysis uncovered that the KTC1-VEM2 subpopulation acquired a point mutation in codon 12 (G12D, c.35G > A) of KRAS, which was not found in the other long-term treated cells or original KTC1 line by Sanger sequence analysis. This acquired mutation was confirmed in Western blots using an antibody specific for the KRAS G12D mutation (Figure 2A). Knockdown of KRAS using siRNA confirmed the specificity of the antibody; scrambled and BRAF-specific siRNA were used as controls. Additionally, we performed Droplet Digital™ PCR (ddPCR) and calculated that 50% of cells in the KTC1-VEM2 subpopulation harbored the KRAS G12D mutation at passage 21 of long-term vemurafenib treatment (Table 2). The BRAF V600E mutation was found in 100% of all cell lines and KTC1 subpopulations used in this study. The G12D point mutation renders KRAS insensitive to the actions of GTPase-activating proteins, locking KRAS in an active state [19]. Moreover, activating RAS mutations have been associated with poorly differentiated disease [20–23]. Consistent with this association, expression of the thyroid follicular cell differentiation markers *PAX8* and *NKX2-1* was decreased in our BRAF/KRAS-mutant cells (KTC1-VEM2) compared with control cells (Figure 2B). In the KTC1-VEM1 cells, only *PAX8* marker was decreased.

**Table 1:** Genetic loci profile of KTC1 subpopulations

	Genetic STR Loci Profile													
Cell line/Subpopulation	AMEL	CSF1PO	D13S317	D16S539	D18S51	D21S11	D3S1358	D5S818	D7S820	D8S1179	FGA	TH01	TPOX	vWA
BCPAP	X	13	12	11,12	17	30,31.2	16,17	10,11	10	12,13	20,23	6,9.3	8,11	14,17
KTC1	X,Y	10,12	11,12	12	12,13	29	14,15	11,12	11	11,14	23,26	9	11	14,17
KTC1-DMSO	X,Y	10,12	11,12	12	12,13	29	14,15	11,12	11	11,14	23,26	9	11	14,17
KTC1-VEM1	X,Y	10,12	11,12	12	12,13	29	14,15	11,12	11	11,14	23,26	9	11	14,17
KTC1-VEM2	X,Y	10,12	11,12	12	12,13	29	14,15	11,12	11	11,14	23,26	9	11	14,17

KTC1 subpopulation identities are matched to the original KTC1 cell line and compared with the BCPAP cell line using short tandem repeat (STR) DNA fingerprinting

**Table 2:** Fractional abundance of BRAF V600E and KRAS G12D mutations

Sample	Gene Target	Mutation Copy #	WT Copy #	FA	Mutation Status
BCPAP	BRAF V600E & WT	3081	0	100.0%	hemizygous
KTC1	BRAF V600E & WT	2134	2242	97.5%	heterozygous
KTC1-DMSO	BRAF V600E & WT	1782	1820	98.9%	heterozygous
KTC1-VEM1	BRAF V600E & WT	658	648	100.8%	heterozygous
KTC1-VEM2	BRAF V600E & WT	2448	2403	100.9%	heterozygous
BRAF NTC	BRAF V600E & WT	0	16704	0.0%	
BCPAP	KRAS G12D & WT	1	4656	0.0%	WT
KTC1	KRAS G12D & WT	1	5048	0.0%	WT
KTC1-DMSO	KRAS G12D & WT	0	4323	0.0%	WT
KTC1-VEM1	KRAS G12D & WT	1	6493	0.0%	WT
KTC1-VEM2	KRAS G12D & WT	1414	4265	49.8%	heterozygous
KRAS NTC	KRAS G12D & WT	0	17125	0.0%	

Droplet Digital™ PCR (ddPCR) was used to determine the fractional abundance (FA) of cells with wild-type (WT) BRAF and mutant BRAF V600E, as well as WT KRAS and mutant KRAS G12D, in the original KTC1 cell line and KTC1 subpopulations. The FA of cells with the BRAF V600E mutation was calculated based on the mutational status previously published for the two original papillary thyroid cancer (PTC) cell lines, BCPAP (hemizygous) and KTC1 (heterozygous). The FA of KTC1-VEM2 cells with KRAS G12D was calculated based on the assumption that the cells were heterozygous for the mutation. No template controls (NTCs) were used in both gene target assays.

### Long-term vemurafenib-treated KTC1 subpopulations exhibit different proliferative behaviors and responses to pathway inhibitors

**Figure 3:**
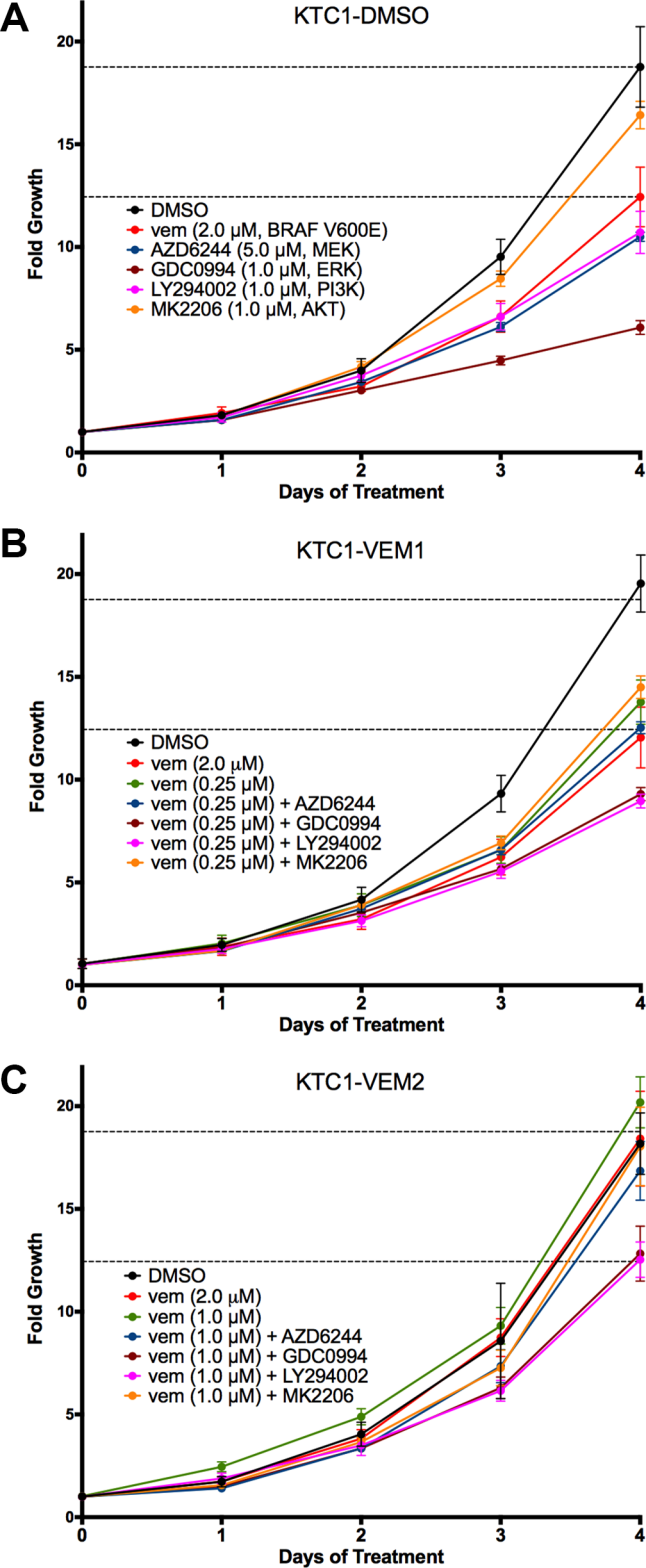
Four-day growth curves performed on KTC1 subpopulations (A) KTC1-DMSO; (B) KTC1-VEM1; (C) KTC1-VEM2) with and without PI3K/AKT and RAF/MEK/ERK pathway inhibitors Cells were treated on day 0 with either no inhibitor (DMSO only), vemurafenib alone (2.0 μM), the vemurafenib concentration each subpopulation was exposed to during long-term passaging (KTC1-DMSO, none; KTC1-VEM1, 0.25 μM; and KTC1-VEM2, 1.0 μM), or vemurafenib in combination with kinase inhibitors for AKT (1.0 μM MK2206), PI3K (1.0 μM LY294002), MEK (5.0 μM AZD6244), and ERK1/2 (1.0 μM GDC0994). Points represent fold growth each day of treatment normalized to cell number counted just prior to treatments for each subpopulation. Dotted lines represent DMSO and 2.0 μM vemurafenib treatments of the KTC1-DMSO cells on each figure.

**Figure 4:**
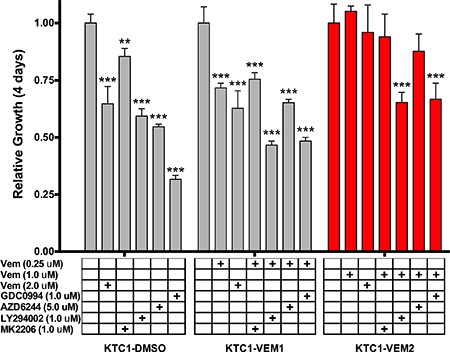
Comparison of relative growth after 4 days of inhibitor treatments (described in Figure 3) Sensitivity to kinase inhibitors is either lost or diminished in the BRAF V600E/KRAS G12D mutant cells (KTC1-VEM2, red bars) in comparison to the untreated cells (KTC1-DMSO) and the long-term vemurafenib-treated cells without the acquired KRAS mutation (KTC1-VEM1).

Four-day growth assays were performed for each KTC1 subpopulation using targeted inhibitors for BRAF V600E (vemurafenib, 2.0 μM), AKT (MK2206, 1.0 μM), PI3K (LY294002, 1.0 μM), MEK (AZD6244, 5.0 μM), and ERK1/2 (GDC0994, 1.0 μM). Fold growth, relative to day zero, for each of the 4 days of treatment is represented by growth curves (Figure 3A–3C). For the long-term vemurafenib-exposed cells, KTC1-VEM1 and KTC1-VEM2, inhibitors were used in combination with the selected vemurafenib dosage (0.25 μM or 1.0 μM). Data for day 4 of the proliferation assays relative to the control DMSO treatment of each of the KTC1-DMSO, KTC1-VEM1, and KTC1-VEM2 cell subpopulations are summarized in Figure 4. As expected, the BRAF V600E, AKT, PI3K, MEK, and ERK1/2 inhibitors significantly decreased the growth of the KTC1-DMSO cells by 35.3%, 14.5%, 40.7%, 45.3%, and 68.4%, respectively, compared with control cells without inhibitors. The KTC1-VEM1 cells were still sensitive to the BRAF V600E inhibitor, with 2.0 μM vemurafenib decreasing growth by 37.2% and 0.25 μM vemurafenib decreasing growth by 28.3%, compared with control cells without inhibitors. Treatment of the KTC1-VEM1 cells with AKT, PI3K, MEK, and ERK1/2 inhibitors in combination with vemurafenib (0.25 μM) decreased growth by 24.5%, 53.4%, 34.8%, and 51.6%, respectively. In the case of the BRAF/KRAS-mutant cells (KTC1-VEM2), our data showed a complete loss of sensitivity to vemurafenib at concentrations of 1.0 μM or 2.0 μM. However, growth was significantly decreased by vemurafenib (1.0 μM) in combination with PI3K and ERK1/2 inhibitors following 4-day treatments, by 34.7% and 33.2%, respectively. The small proliferation decrease seen with the addition of MEK inhibitor (12.2%) was not statistically significant. In contrast, KTC1-DMSO and KTC1-VEM1 cells were both responsive to MEK inhibitor, with 45.3% and 34.8% reductions, respectively.

Pathway activation 4 and 24 hours after ERK1/2 and MEK inhibitor treatment was also quantified from Western blots. The ERK1/2 inhibitor (GDC0994) significantly decreased ERK1/2 activation at 4 hours in all three subpopulations compared to control treatments (Supplementary Figure 2). Levels of activated ERK1/2 recovered after 24 hours of GDC0994 treatment in all three subpopulations; however, activated ERK1/2 remained relatively higher in the KTC1-VEM1 and KTC1-VEM2 cells compared to the KTC1-DMSO cells. Compared with the control treatments, 24-hour treatment with the MEK inhibitor (AZD6244) maintained significantly decreased levels of activated ERK1/2 in all three subpopulations of cells.

**Figure 5:**
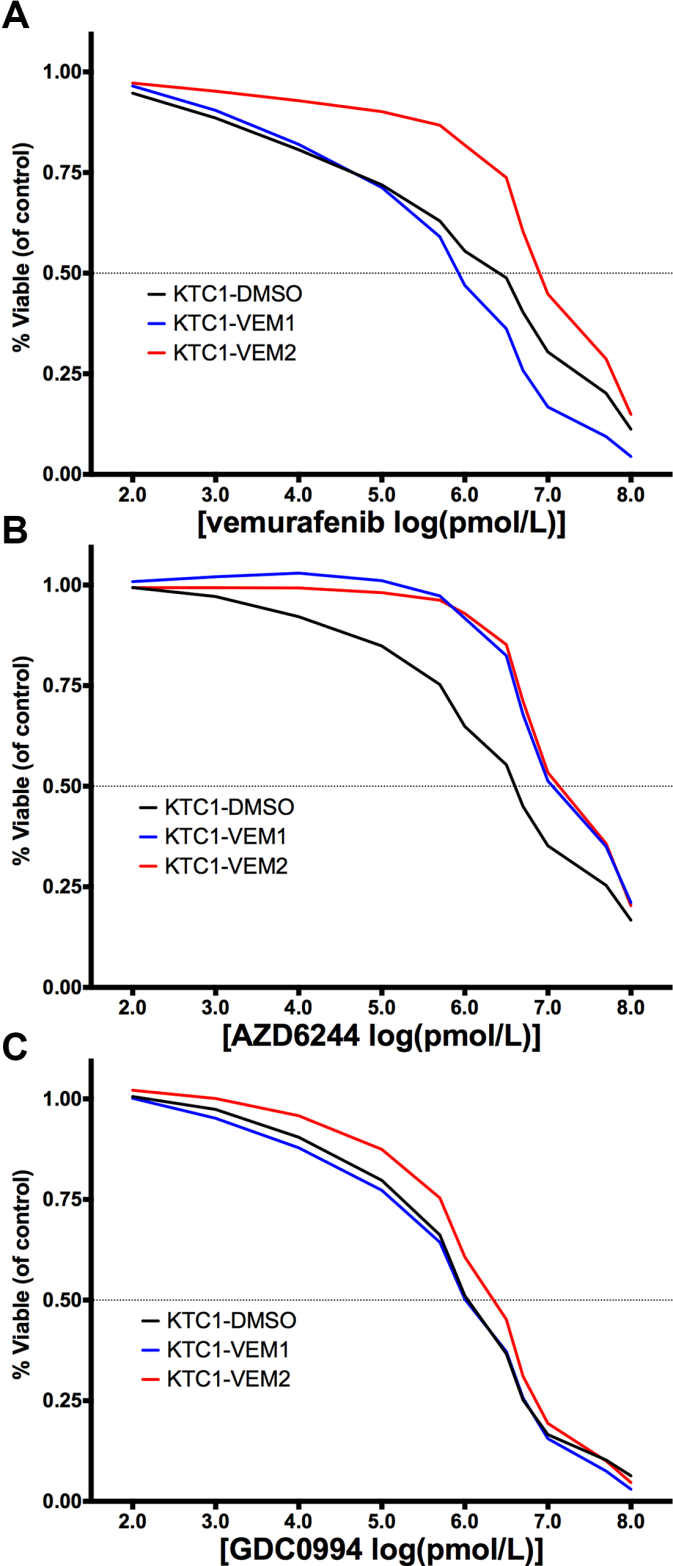
Dose-response curves (percentage of treated cell number compared with DMSO-treated control) for KTC1 subpopulations treated for 4 days with (A) vemurafenib, a BRAF V600E inhibitor; (B) AZD6244, a MEK inhibitor; and (C) GDC0994, an ERK/1/2 inhibitor at the doses described in Supplementary Table 3 The x-axis represents the log-transformed inhibitor dose concentration in pmol/L. The BRAF V600E/KRAS G12D mutant cells are displayed as red lines.

Since the KTC1-VEM2 cells demonstrated no sensitivity to vemurafenib at concentrations as high as 2.0 μM, dose-response experiments were performed to assess the IC50 values of the three KTC1 subpopulations. The vemurafenib IC50 value, with 95% confidence range in parentheses, for the KTC1-VEM2 cells was 7.4 μM (5.1–10.1 μM), which was 4-fold higher than the IC50 value for the KTC1-DMSO cells, 1.9 μM (0.8–4.6 μM), and 7-fold higher than the IC50 values for the KTC1-VEM1 cells, 1.1 μM (0.6–2.0 μM) (Figure 5A). MEK and ERK1/2 are downstream of the RAF family members along the RAS/RAF/MEK/ERK kinase cascade and thus are of interest as inhibition targets in many cancers. Therefore, we also ran dose-response experiments using the MEK inhibitor AZD6244 and the ERK1/2 inhibitor GDC0944. We observed 5- and 4-fold increases in AZD6244 IC50 values for the KTC1-VEM2 and KTC1-VEM1 cells compared with KTC1-DMSO; the values were 10.2 μM, 8.9 μM, and 2.1 μM, respectively (Figure 5B). There were no meaningful differences between the GDC0994 IC50 values among the KTC1-DMSO, KTC1-VEM1, and KTC1-VEM2 cells, which were 1.1 μM, 1.2 μM, and 1.8 μM, respectively (Figure 5C).

### KTC1-VEM2 cells utilize activated KRAS to bypass BRAF V600E inhibition

**Figure 6:**
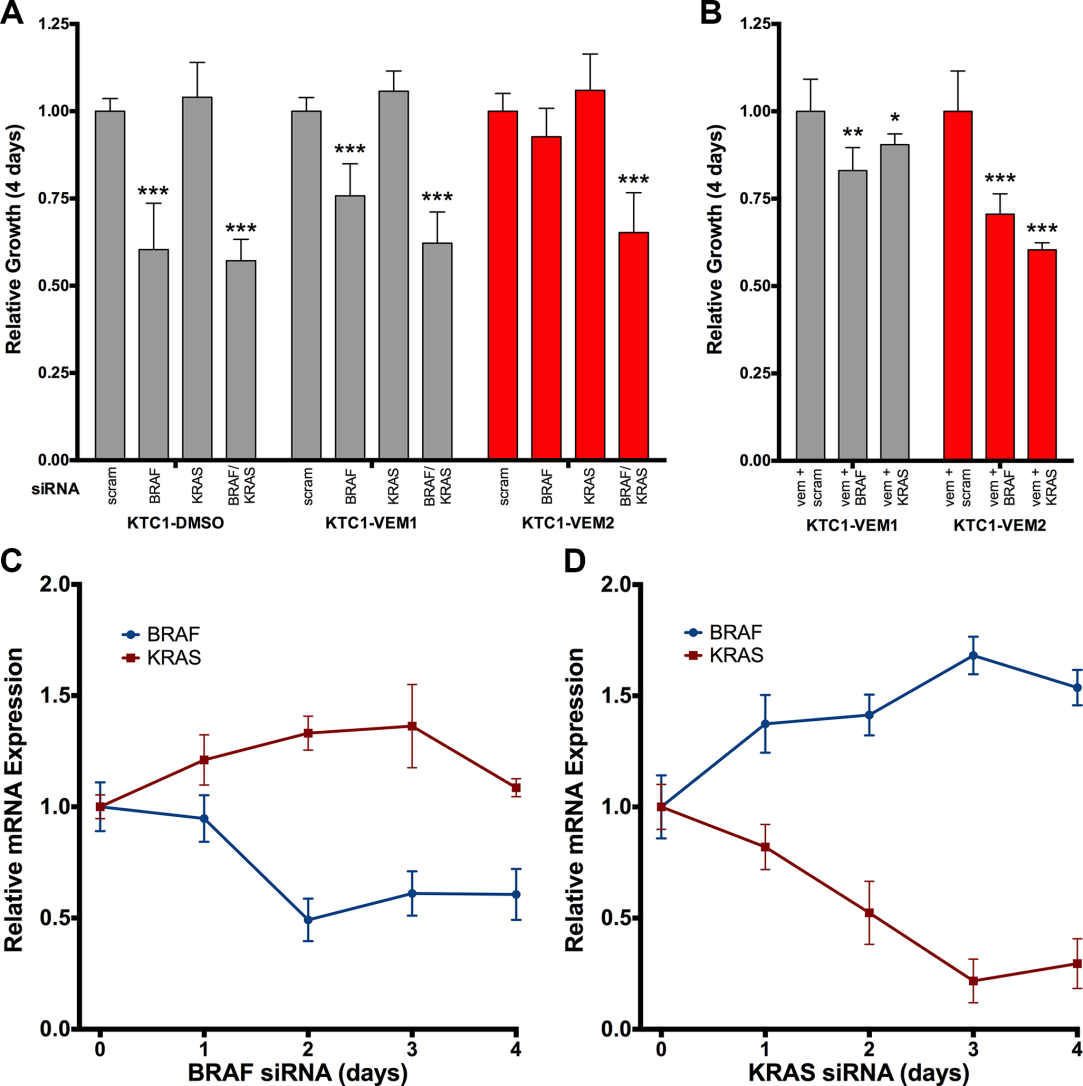
Four-day proliferation analysis of KTC1 subpopulations following siRNA knockdowns of BRAF and KRAS (**A**) siRNA knockdown of both BRAF and KRAS, but not either individually, decreases the growth of the BRAF V600E/KRAS G12D mutant cells (KTC1-VEM2, red bars). KRAS siRNA alone has no effect on any subpopulation. (**B**) siRNA knockdown of either BRAF or KRAS in the presence of 2.0 μM vemurafenib does decrease proliferation of the KTC1-VEM2 cells. Growth is relative to scrambled siRNA controls of each subpopulation. (**C** and **D**) Relative BRAF and KRAS transcript expression in the KTC1-VEM2 cells throughout the 4-day siRNA proliferation experiment shows a compensatory expression effect resulting from the knockdown of each molecule. mRNA expression is normalized to scrambled siRNA levels for each day and relative to day 0.

To confirm that mutant KRAS activation drives vemurafenib resistance in KTC1-VEM2 cells, we performed KRAS and BRAF knockdowns in the three subpopulations and measured their rate of proliferation over 4 days. The KTC1-DMSO and KTC1-VEM1 cells displayed BRAF-dependent cell growth; knockdowns significantly decreased proliferation by 39.6% and 24.2%, respectively (Figure 6A). Proliferation of the KTC1-VEM2 cells was not inhibited by individual BRAF or KRAS knockdowns, which would be expected if these driver mutations worked in a compensatory manner. Congruent with this compensatory effect, dual knockdowns of BRAF and KRAS or KRAS knockdown in combination with vemurafenib significantly decreased proliferation by 34.7% and 39.6% compared with control (Figure 6A, 6B). Interestingly, vemurafenib treatment combined with a knockdown of BRAF was sufficient to significantly decrease growth by 29.4% in the KTC1-VEM2 cells (Figure 6B). This result could mean that the KRAS G12D mutation alone is not sufficient to completely drive proliferation in these cells. To investigate this further, we assessed whether compensatory expression changes were occurring between KRAS and BRAF during knockdown experiments in these cells. We evaluated KRAS and BRAF transcript levels in the KTC1-VEM2 cells throughout the progress of the knockdown experiments. Figure 6C and 6D show that knocking down BRAF or KRAS proportionally increased expression of the other pathway activator over time relative to scrambled siRNA, effectively compensating for the reduction in pathway activators in the KTC1-VEM2 cells not treated in adjunct with vemurafenib. However, individual siRNA knockdown combined with vemurafenib did result in significant decreases in ERK1/2 activation (Supplementary Figure 3), which correlated to the decreased growth observed for this treatment combination in these cells (Figure 6B).

### Expression and activation of receptor tyrosine kinases are increased in the KTC1 subpopulations

**Figure 7:**
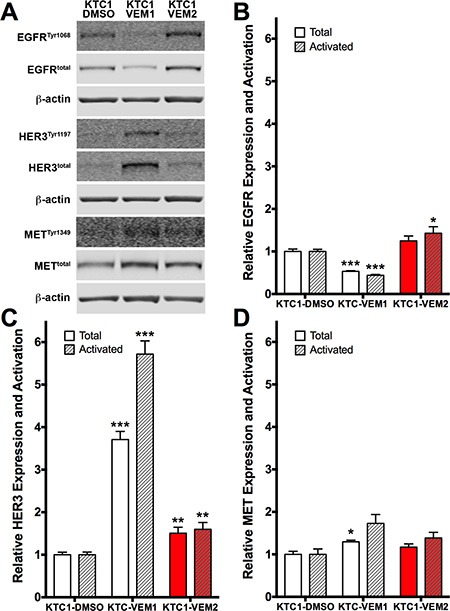
Receptor tyrosine kinase (RTK) expression and activation in KTC1 subpopulations following long-term exposure to DMSO (KTC1-DMSO) or vemurafenib (KTC1-VEM1 and KTC1-VEM2) (**A**) Western blots probing for activated and total EGFR, HER3, and MET. Quantification of (**B**) EGFR, (**C**) HER3, and (**D**) MET bands (normalized to β-actin) shows increased levels of activated EGFR as well as total and activated HER3 in the BRAF V600E/KRAS G12D mutant cells (KTC1-VEM2, red bars) compared with the control cells (KTC1-DMSO). Total and activated forms of HER3 are increased, whereas EGFR is decreased, in the KTC-VEM1 cells.

Activation of receptor tyrosine kinases (RTKs), such as HER3, has been demonstrated to be a primary resistance mechanism in thyroid cancer cell lines following short-term culture (2–4 days) [13]. Therefore, we performed Western blotting to compare expression and activation levels of three relevant RTKs in our KTC1 subpopulations. Our data demonstrate that each subpopulation has a unique expression and activation profile for these three RTKs (Figure 7A). KTC1-VEM1 cells expressed significantly lower levels of total EGFR, 46.7% less, and active EGFR, 56.0% less, compared with KTC1-DMSO cells (Figure 7B). However, expression and activation of HER3 in KTC1-VEM1 cells were significantly higher, 3.7- and 5.7-fold, respectively (Figure 7C). This decrease in EGFR and increase in HER3 activity are similar to levels previously described for thyroid cancer cell lines in response to short-term vemurafenib treatment [13]. Levels of total and activated HER3 were significantly increased in the BRAF/KRAS-mutant cells, KTC1-VEM2, but to a lesser degree, 1.5- and 1.6-fold, respectively, compared with KTC1-DMSO cells (Figure 7C). Interestingly, EGFR activation significantly increased by 1.4-fold over levels observed in the untreated KTC1-DMSO cells, and by 3.2-fold over levels in the KTC1-VEM1 cells treated with 0.25 μM vemurafenib (Figure 7B). MET expression and activation were slightly higher in both the KTC1-VEM1 and KTC1-VEM2 cells compared with the control cells, but only total MET in the KTC1-VEM1 cells demonstrated a statistically significant change.

### KTC1 subpopulations have different steady-state activation levels of the PI3K and MAPK pathways

**Figure 8:**
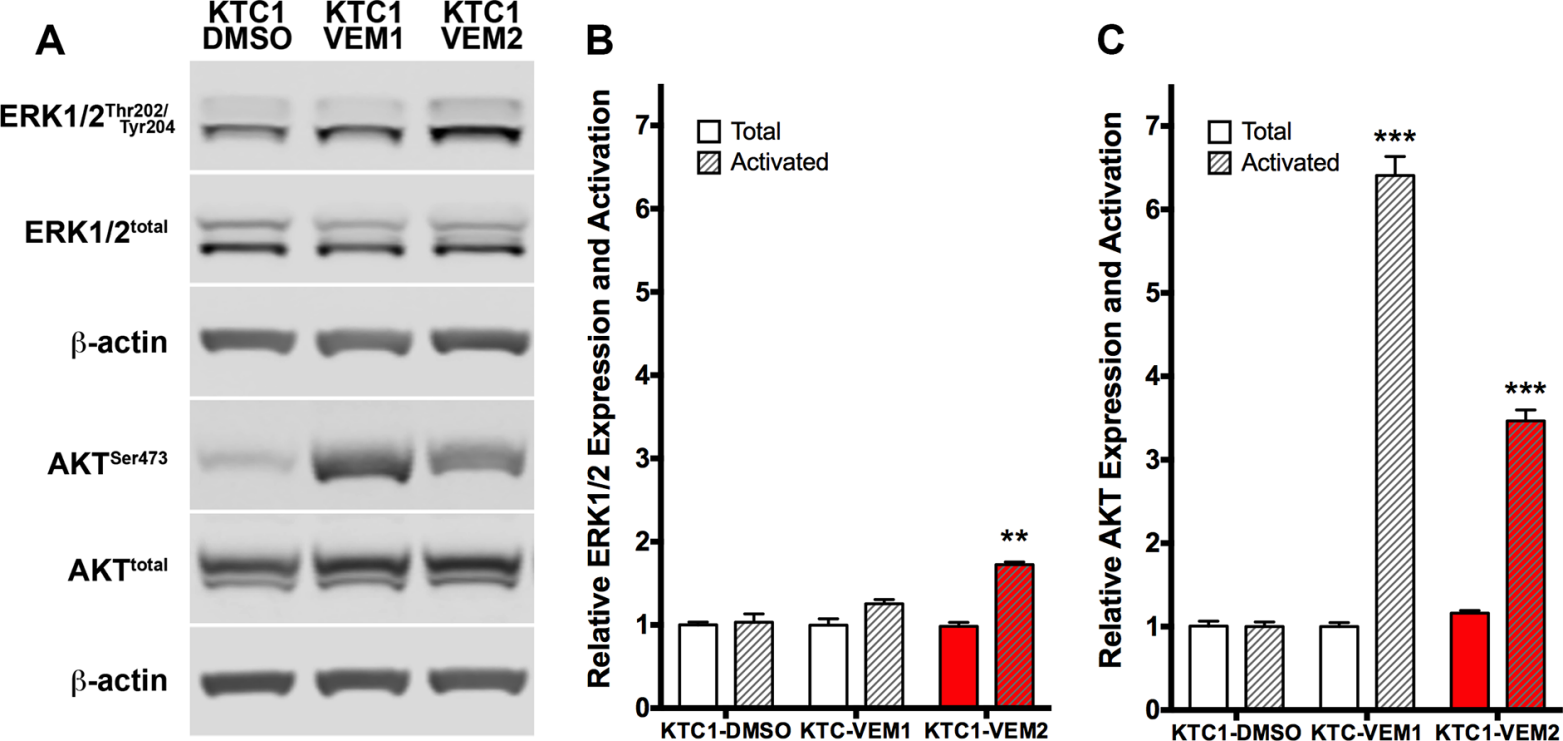
PI3K/AKT and RAF/MEK/ERK pathway activation in KTC1 subpopulations following long-term exposure to DMSO (KTC1-DMSO) or vemurafenib (KTC1-VEM1 and KTC1-VEM2) (**A**) Western blots probing for activated and total ERK1/2 and AKT. Quantification of (**B**) ERK1/2 and (**C)** AKT bands (normalized to β-actin) shows activation of only the PI3K/AKT pathway in the KTC1-VEM1 cells but activation of both PI3K/AKT and RAF/MEK/ERK pathways in the BRAF V600E/KRAS G12D mutant cells (KTC1-VEM2, red bars) compared with the control cells (KTC1-DMSO).

The mechanism of inhibitor resistance drove a significant shift in the signaling pathways used by each of the KTC1 subpopulations to proliferate in the presence of vemurafenib. Western blot analysis showed that KTC1-VEM1 cells predominantly activate AKT and not ERK signaling, which may contribute to the subpopulation's survival in continual culture with vemurafenib (Figure 8A–8C). Levels of activated AKT in the KTC1-VEM1 cells significantly increased, by 6.4- fold, in comparison to KTC1-DMSO cells (Figure 8C). Conversely, the PI3K/AKT/mTOR and RAS/RAF/MEK/ERK pathways were activated in the KTC1-VEM2 cells containing the BRAF and KRAS mutations. Levels of activated AKT significantly increased by 3.5- fold, while activated ERK1/2 significantly increased by 1.8- fold compared with KTC1-DMSO cells (Figure 8B). Total kinase levels did not significantly change in either subpopulation of cells. Taken together, these data show how individual or combined activation of these pathways leads to inhibitor resistance.

## DISCUSSION

Although most patients diagnosed with PTC have excellent long-term outcomes after primary surgical therapy, the presence of an oncogenic BRAF V600E mutation is associated with refractory disease and worse clinical outcomes. Therefore, efforts have been directed towards use of selective BRAF V600E inhibitors in thyroid cancers as systemic therapies. Although the BRAF V600E inhibitors vemurafenib and dabrafenib have shown some promise in clinical trials, response is short lived, acquired inhibitor resistance ultimately develops, and overall survival may not be improved. For that reason, research to improve clinical outcomes has been recently focused on elucidating possible mechanisms of resistance to BRAF V600E inhibitors. However, to date, most translational experiments trying to elucidate mechanisms of resistance to BRAF inhibition have involved short-term *in vitro* studies. These studies described activation of survival pathways through response mechanisms that might reflect primary resistance and did not describe acquisition of secondary resistance mechanisms that may result from selective pressures through chronic inhibitor treatment.

Our study identified a subpopulation of BRAF V600E PTC cells that acquired a novel KRAS G12D mutation following long-term inhibitor pressure. This mutation conferred adaptive resistance to vemurafenib as well as other kinase inhibitors, such as a MEK inhibitor. The resulting amino acid substitution, 12G > D, impairs intrinsic GTP hydrolysis and GTP hydrolysis mediated by GTPase-activating protein, which leads to constitutive activation of KRAS [24, 25]. KRAS G12D is predominant in pancreatic ductal adenocarcinomas, non-small-cell lung cancer, colorectal cancer, and other carcinomas [6, 26–31]. RAS mutations (KRAS, NRAS, and HRAS) are found at a relative low frequency in differentiated thyroid cancers compared with the more common BRAF mutations [32]. but their detection in PTC and follicular thyroid cancers (FTCs) has increased over the last decade and a half [33]. Although the frequency of the specific RAS family member and its coincidence with BRAF V600E are debated, activating RAS mutations have been associated with more aggressive poorly differentiated disease [20–23, 34].

In mouse models of thyroid cancers, tissue-specific knock-in of *Kras^G12D^* leads to follicular cell hyperplasia only. However, concomitant loss of *Pten (Kras^G12D^/Pten* ^− /−^*/TPO-cre)* leads to FTCs, which rapidly progress to poorly differentiated thyroid cancer and anaplastic thyroid cancer (ATC) [35–37]. Mice harboring a potent dominant negative mutant thyroid hormone receptor β, TRβPV (*Thrb^PV/PV^*), spontaneously develop well-differentiated FTCs phenotypically similar to human cancer, and *Thrb^PV/ PV^Kras^G12D^* mice develop frequent anaplastic foci with complete loss of normal thyroid follicular morphology [38]. According to a mutation analysis study of 41 thyroid cancer cell lines, 10 contained RAS activating mutations. However, only two of the 41 RAS-mutant lines were KRAS mutants, and none coexisted with a BRAF mutation [34]. No mouse model has been yet developed with concomitant BRAF V600E and KRAS G12D mutations. Development of ATC in two thyroid cancer patients treated with vemurafenib has been described, but the mechanism by which the ATC transformation occurred was not studied [39]. However, BRAF inhibitors are known to cause malignant transformation in pre-existing RAS-mutated skin lesions [40]. Therefore, RAS-driven acquired resistance causing transformation to ATC or leading to progression of PTC is a plausible mechanism. In an interesting study, Das Thakur and colleagues demonstrated that vemurafenib-resistant melanoma cells became drug dependent for their continued proliferation and that cessation of drug administration led to regression of established drug resistance [41]. The premise of this counterintuitive response is that higher levels of MAPK pathway activation can be toxic to a cell. Based on this premise, it is possible that concurrent KRAS and BRAF mutations without the presence of a BRAF V600E inhibitor could push MAPK pathway activation to such a toxic level. However, the BRAF/KRAS-mutant KTC1-VEM2 cells do not seem to be inhibitor dependent as they grow at similar rates with or without vemurafenib.

**Figure 9:**
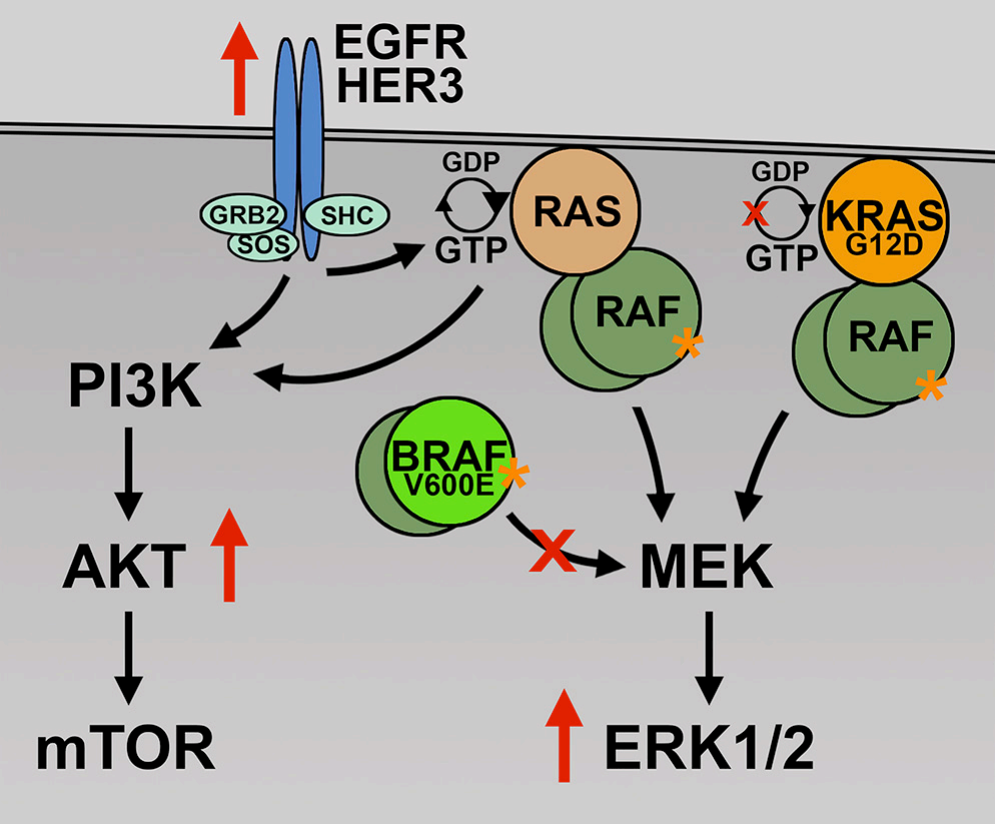
Proposed mechanism of vemurafenib resistance In the presence of BRAF V600E inhibition (large red X), the BRAF/KRAS mutant cells, KTC1-VEM2, are able to drive activation of survival and proliferation pathways (PI3K/AKT and RAF/MEK/ERK) through the KRAS G12D mutation and increased RTK (EGFR and HER3) activation. The KRAS G12D mutation is insensitive to the actions of GTPase-activating proteins (small red X) and is constitutively active (orange asterisk).

The KTC1 subpopulations described in this study provide a model of spontaneously acquired secondary resistance to BRAF V600E inhibitor in thyroid cancer cells. Accordingly, the resulting subpopulations following long-term vemurafenib treatment utilized different survival mechanisms. KTC1 cells with the BRAF V600E mutation and no KRAS mutation (KTC1-VEM1) predominantly used HER3 activation and consequent activation of the PI3K/AKT pathway as an attempt to survive short-term vemurafenib exposure. This response matches previously observed short-term responses of ATC cells (SW1736 and 8505C) harboring only the BRAF V600E mutation [13, 18]. In contrast, our studies suggest that a coexisting KRAS G12D mutation (KTC1-VEM2) may sustain RAS/MEK/ERK signaling concurrent with RTK-mediated PI3K/AKT pathway activation (Figure 9). This concurrent pathway activation was reported in other studies identifying BRAF inhibitor resistance mechanisms in melanoma, where enhanced signaling through the PI3K/AKT pathway is often observed in addition to adaptive reactivation of the MAPK pathway [42–45].

Mechanistically, activating KRAS mutations sustain RAS/MEK/ERK signaling using CRAF as an alternative downstream receiver [46–48]. Lito and colleagues recently demonstrated that MEK activated by CRAF in melanoma and lung cancer cells is less sensitive to MEK inhibitors than when activated by BRAF V600E [49], which is confirmed by the response of KTC1-VEM2 cells to combination treatment with vemurafenib and the MEK inhibitor AZD6244. However, our data showed only a marginally significant decrease in KTC1-VEM2 cell growth and no significant difference in KTC1-VEM1 cell growth, when compared to KTC1-DMSO cells, in response to CRAF knockdown (Supplementary Figure 4). Interestingly, the KTC1 heterogeneous subpopulation expressing KRAS G12D was more resistant to BRAF V600E, MEK, and ERK inhibitor treatment. Knockdown of BRAF and KRAS using siRNA had an additive effect in decreasing cell proliferation in response to vemurafenib. Taken together, these functional and biochemical studies support KRAS G12D as a mechanism of BRAF inhibitor resistance in PTC. However, clinical correlation will be necessary to predict those patients with intrinsic resistance to vemurafenib and definitively demonstrate the utility of KRAS G12D hotspot mutation in predicting intrinsic resistance to BRAF inhibitors.

The aberrant oncogenic signaling caused by the KRAS G12D hotspot mutation in PTC, and potential combination therapies to target this mutation, remain an important area of investigation for future study. However, as KRAS is widely assumed to be undruggable, identifying other critical downstream targets and effector pathways that induce PTC cell proliferation and/or invasion will be necessary to improve future treatments.

## MATERIALS AND METHODS

### Original cell lines

The KTC1 cell line was originally derived from the pleural effusion of a 68-year-old male PTC patient [50] and harbors a heterozygous BRAF V600E mutation [18]. The BCPAP cell line was originally derived from the primary tumor of a 76-year-old woman with PTC with local and lymph node metastasis [51]. and it is homo/hemizygous for the BRAF V600E mutation [18]. Both lines were kindly provided by Dr. R. Schweppe, Department of Medicine, Division of Endocrinology, University of Colorado, Denver, CO, with a passage number status of p20 for each. Cells were cultured in DMEM/F12 medium (Thermo Fisher Scientific, Waltham, MA) supplemented with 10% fetal bovine serum (Sigma-Aldrich, St. Louis, MO).

### Creation of KTC1 subpopulations

Separate flasks of KTC1 cells were cultured long-term for 20 further passages (20 weeks) in the continual presence of DMSO (KTC1-DMSO), 0.25 μM vemurafenib (KTC1-VEM1), or 1.0 μM vemurafenib (KTC1-VEM2) delivered with a final DMSO concentration of 0.03% in medium (Supplementary Figure 1). The overall passage number status of these subpopulations of cells following the long-term vemurafenib treatment was p40 from their original line derivation. The cell lines’ genetic identity was confirmed with STR fingerprinting following the long-term vemurafenib treatment.

### Mutation analysis

Mutation analysis was performed by the Characterized Cell Line Core facility (The University of Texas MD Anderson Cancer Center) using a Sequenom MALDI TOF Mass Array system to assay for a panel of 420 common somatic mutations (Supplementary Table 2). Confirmation of the KRAS G12D mutation was performed through Droplet Digital^™^ PCR and Western blotting using a rabbit monoclonal antibody specific for the KRAS G12D mutation (Cell Signaling, #14429).

### STR fingerprinting

Cell line identity was validated by the MD Anderson Characterized Cell Line Core facility (supported by the NIH/NCI Cancer Center Support Grant, CA016672) with STR DNA fingerprinting, which used the Promega 16 High-Sensitivity STR Kit (Catalog # DC2100). The STR profiles were compared with online search databases (DSMZ/ATCC/JCRB/RIKEN) of 2455 known profiles and with the MD Anderson Characterized Cell Line Core database of 2556 known profiles. The KTC1 cell line STR profile was matched with a previously published profile [18].

### Cell proliferation assays

Equal numbers of cells (~1000) were plated and grown in five 96-well plates containing DMEM/F12 supplemented with 10% serum and the inhibitors or siRNA described below. One plate was collected and fixed (4% PFA) prior to treatment (day 0) to be used to determine the starting number of cells for each cell line or subpopulation. A plate was then collected and fixed on each day (days 1–4) following treatments with either pharmacological inhibitors or targeted siRNA. All plates were stained with the nuclear dye DAPI just prior to tile imaging of entire wells using a high-throughput IN Cell Analyzer (GE Healthcare Life Sciences) kindly made available by the High-Throughput Screening Core Lab, Institute for Biosciences and Technology, Texas A&M Health Science Center (Houston, TX). Images were then stitched together and counted using the system's image analysis software. Fold growth was determined for each day from the average cell numbers contained in the wells of a treatment group (8 to 12 wells per cell line and treatment) and normalized to the starting cell number (average day 0 values). Relative fold growth was calculated from fold growth on day 4 of treatment normalized to fold growth of control-treated cells on day 4.

### Pharmacological inhibitors

Cell proliferation assays were performed as stated above using BRAF V600E inhibitor (vemurafenib), ERK1/2 inhibitor (GDC0994), MEK inhibitor (AZD6244, selumetinib), and pan-AKT inhibitor (MK2206) purchased from Selleck Chemicals (Houston, TX). Stock solutions of each inhibitor were prepared in 100% DMSO (Sigma, St Louis, MO). Final stated inhibitor concentrations were delivered in media at a 1:3000 (v/v) ratio, giving a final DMSO concentration of 0.03% in experiments containing only single inhibitors and 0.06% in experiments that contained combinations of two inhibitors. Equivalent DMSO concentrations served as vehicle controls in each.

### Dose-response curves

Cell proliferation assays were performed as stated above using inhibitor concentrations of 0.0001, 0.01, 0.1, 0.5, 0.75, 1.0, 2.5, 5.0, 10.0, 50.0, and 100.0 μM. Log-transformed dose-response curves were drawn using a 6th order smoothing polynomial function in Prism software, version 6.0e. (GraphPad Software, Inc., La Jolla, CA). IC50 values and significance were calculated from these curves using a three-parameter inhibitor dose-response analysis function available in the Prism software. Supplementary Table 3 contains the relative dose-response curve values for each inhibitor used to plot the curves.

### RNA interference

Cell proliferation assays were performed as stated above using commercially available siRNA for *BRAF*, *KRAS*, and a negative control (#4392420, #4390824, and #4390843, Thermo Fisher Scientific, Waltham, MA). siRNA was transfected into KTC1 subpopulations using Lipofectamine™ RNAiMax according to the manufacturer's guidelines. Serum-free media containing siRNA was changed for fresh media containing 10% serum following overnight incubation. Collection of cell lysates following siRNA treatment was performed in triplicate in 6-well plates for Western blot analysis, whereas collection of mRNA was performed in triplicate in 96-well plates for quantitative real-time PCR.

### RNA extraction and quantitative real-time PCR

RNA extraction, reverse transcription, and qRT-PCR were performed using the TaqMan^®^ Gene Expression Cells-to-CT Kit (Thermo Fisher Scientific, Waltham, MA) according to the manufacturer's guidelines. Relative fold transcript change was determined using the ΔΔCq method. The TaqMan^®^ probe assays used for specific transcripts are *BRAF* (Hs00269944_m1), *KRAS* (Hs00364284_g1), *PAX8* (Hs01015257_g1), *NKX2-1* (Hs00968940_m1), and *ACTB* (Hs01060665_g1).

### Western blot analysis

Proteins were isolated from cell cultures after described treatments using lysis buffer containing protease and phosphatase inhibitors, then run on 4–12% polyacrylamide gels (BioRad, Hercules, CA) and blotted on nitrocellulose membranes according to standard protocols. After transfer, blots were blocked with 5% milk in Tris-buffered saline (TBS) for 1 hour at room temperature and then probed overnight at 4°C with primary antibodies diluted to the appropriate concentrations and diluent (Supplementary Table 4). Blots were incubated with infrared fluorophore conjugated secondary antibodies diluted to the appropriate concentrations in 5% milk in TBS with 0.1% Tween 20 for 1 hour at room temperature (Supplementary Table 4). Fluorescent bands were revealed using an Odyssey^®^ Fc Imager (LI-COR Biotechnology, Lincoln, NE). Band intensities were measured using Odyssey Infrared Imaging System Application Software (v3.0.30), then standardized over β-actin band intensities probed for on the same blot. Standardized intensities were compared with standardized intensity of the DMSO control for relative quantifications. Cell culture experiments were repeated at least twice, probing samples from each experiment on at least two different blots.

### Droplet Digital™ PCR

Quantitative analysis of the mutation allelic frequencies was performed using the QX200™ Droplet Digital™ PCR system (Bio-Rad Laboratories, Hercules, CA). Reactions were performed according to the manufacturer's recommended protocol using PrimePCR™ validated assays for detection of human BRAF p.V600E mutations (dHsaCP2000027 and dHsaCP2000028) and KRAS p.G12D (dHsaCP2000001 and dHsaCP2000002). A master mix of 2× Supermix for Probes (Bio-Rad), 20× wild-type and mutant gene assays (BRAF p.V600E or KRAS p.G12D), 50 ng of DNA, and water was prepared to a total of 22 ml for each sample reaction in sample plates for the Automated Droplet Generator (Bio-Rad Laboratories, Hercules, CA) following the manufacturer's protocol. Each assay included DNA from the RKO (BRAF V600E +) and HT-29 cell lines (KRAS G12D +) as positive controls and no-template controls. Droplets were generated using a Bio-Rad Automated Droplet Generator where Droplet Generation Oil for Probes (Bio-Rad) was added to each reaction mix. Droplets (40 ml total) were then transferred to a 96-well plate and then thermal-cycled with the following conditions: 95°C for 10 minutes, 40 cycles of 94°C for 30 seconds, 55°C for 1 minute, followed by 98°C for 10 minutes. A Bio-Rad Droplet Reader and QuantaSoft Software were used for droplet reading and analysis (Bio-Rad). Fractional abundance (FA) was calculated using FA = (NMut/(NMut + NWT))x2 for heterozygous mutants and FA = NMut/(NMut + NWT) for hemizygous mutants, where NMut is number of mutant events and NWT is number of wild-type events per reaction.

### Statistical analysis

All data were graphed using Prism software and are presented as mean ± standard error of the mean. Two-tailed Student *t*-test was used to compare control and treated groups. For all comparisons, statistical significance was assigned at *P* < 0.05.

## SUPPLEMENTARY MATERIALS FIGURES AND TABLES




