# Supplementary material for: Long-term vemurafenib treatment drives inhibitor resistance through a spontaneous KRAS G12D mutation in a BRAF V600E papillary thyroid carcinoma model
Source: Oncotarget. 2016 Apr 26;7(21):30907–23. doi: 10.18632/oncotarget.9023 (PMC5058727; doi:10.18632/oncotarget.9023)
Supplement: Supplementary file 1 [file oncotarget-07-30907-s001.pdf]

Long-Term vemurafenib treatment drives inhibitor resistance through a spontaneous KRAS G12D mutation in a BRAF V600E papillary thyroid carcinoma model

Supplementary Materials

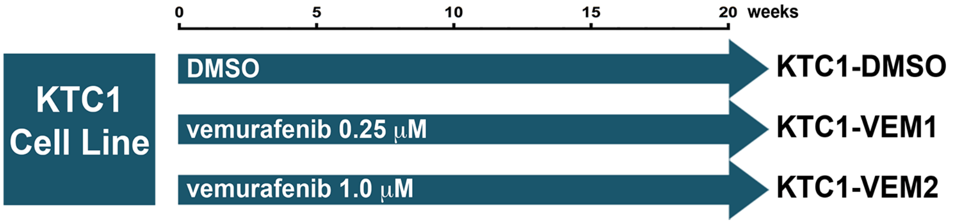

Supplementary Figure S1: Schematic of the long-term treatments used to create the KTC1 subpopulations in this study.

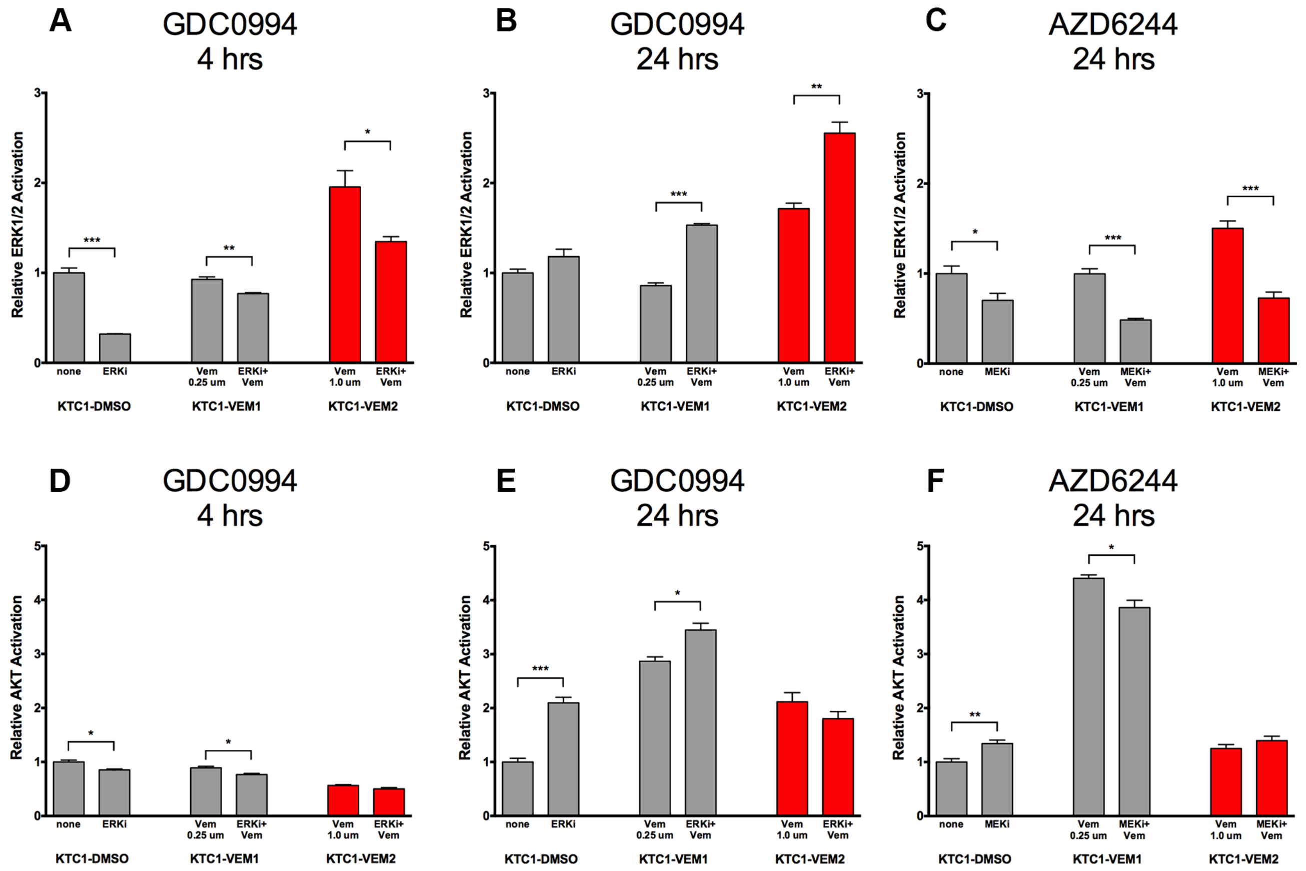

Supplementary Figure S2: Pathway activation after ERK1/2 and MEK inhibitor treatment. KTC1 subpopulations were treated with inhibitor combinations as described in the text, and cell lysates were collected following 4 and 24 hours after ERK1/2 activation inhibition (GDC0994) or 24 hours after MEK activation inhibition (AZD6244). Western blots were used to determine total levels of ERK1/2 (**A–C**) and AKT (**D–F**) activation in treated cells relative to control KTC1-DMSO cells.

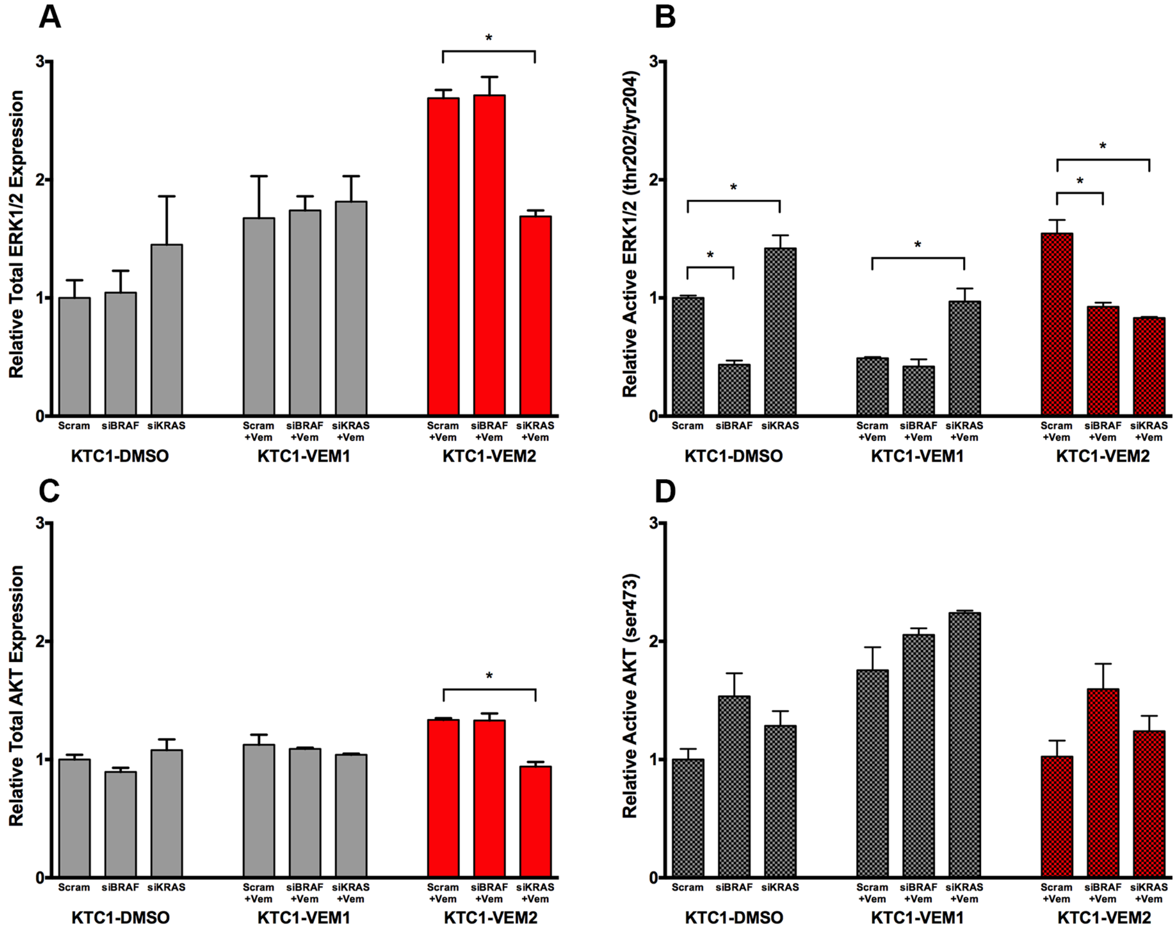

Supplementary Figure S3: Pathway activation in response to BRAF and KRAS mRNA knockdown. KTC1-DMSO cells were treated with BRAF and KRAS siRNA alone. KTC1-VEM1 and KTC1-VEM2 cells were treated with siRNA in combination with vemurafenib, 0.25 μM and 1.0 μM, respectively. Cell lysates were collected 48 hours after transfection. Western blots were used to determine levels of total and activated ERK1/2 (**A, B**) and AKT (**C, D**) in treated cells relative to control KTC1-DMSO cells.

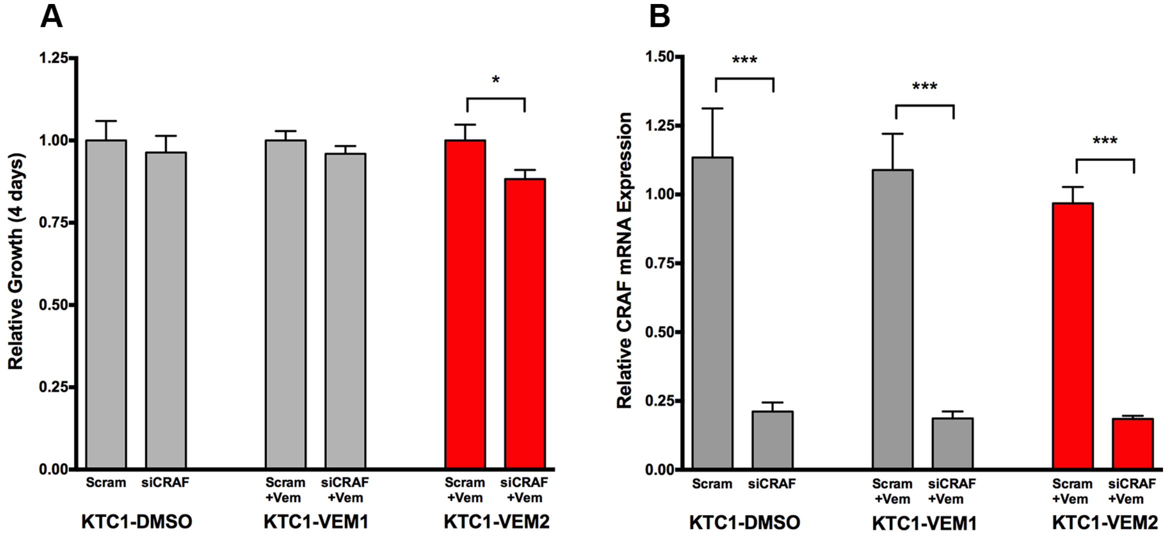

Supplementary Figure S4: Four-day growth assays performed on KTC1 subpopulations following CRAF knockdown. (**A**) KTC1-DMSO cells were treated with CRAF siRNA alone. KTC1-VEM1 and KTC1-VEM2 cells were treated with CRAF siRNA in combination with vemurafenib, 0.25 μM and 1.0 μM, respectively. Western blots were used to determine levels of total and activated ERK1/2 and AKT relative to control KTC1-DMSO cells. Growth is relative to control cells treated with scrambled siRNA for each subpopulation. (**B**) qRT-PCR was used to determine CRAF mRNA knockdown efficiency for each subpopulation.

Supplementary Table S1: Known cancer-associated mutations found in BCPAP and KTC1 cell lines

BCPAP Cell Line Mutations

Gene	AA Mutation	CDS Mutation	Zygosity	Type	Protein Function	References
BIRC3	none	c.954–2A > G	heterozygous	Substitution - Intronic	inhibitor of apoptosis	(Forbes, Beare et al., 2015, Nucleic Acids Research)
BRAF	p.V600E	c.1799T > A	hemizygous	Substitution - Missense	cell proliferation and differentiation	(Schweppe et al., 2008, J Clin Endocrinol Metab, Forbes, Beare et al., 2015, Nucleic Acids Research)
LRP1	p.S2634L	c.7901C > T	heterozygous	Substitution - Missense	tumor invasion	(Forbes, Beare et al., 2015, Nucleic Acids Research)
PXN	p.R431C	c.1291C > T	heterozygous	Substitution - Missense	cell adhesion and motility	(Forbes, Beare et al., 2015, Nucleic Acids Research)
SMAD3	p.D262E	c.786C > A	heterozygous	Substitution - Missense	cell proliferation, differentiation, apoptosis	(Forbes, Beare et al., 2015, Nucleic Acids Research)
TERT	none	c.228C > T	heterozygous	Substitution - Missense	involved in oncogenesis	(Landa et al., 2013, Journal of Clinical Endocrinology & Metabolism)
TP53	p.D259Y	c.775G > T	homozygous	Substitution - Missense	tumor suppressor	(Forbes, Beare et al., 2015, Nucleic Acids Research)

KTC1 Cell Line Mutations

Gene	AA Mutation	CDS Mutation	Zygosity	Type	Protein Function	References
BRAF	p.V600E	c.1799T > A	heterozygous	Substitution - Missense	cell proliferation and differentiation	(Schweppe, Klopper et al., 2008, J Clin Endocrinol Metab)
TERT	none	c.250C > T	heterozygous	Substitution - Missense	involved in oncogenesis	(Landa, Ganly et al., 2013, Journal of Clinical Endocrinology & Metabolism)
MCL1	none	none		copy number gain	pro-survival factor	(Duquette et al., 2015, Oncotarget)
P16	none	none	homozygous	deletion	tumor suppressor	(Duquette, Sadow et al., 2015, Oncotarget)

Supplemental Table S2: Detection primers used and results of Sequemon mutation analysis. See Supplementary_Table_S2

Supplementary Table S3: IC50 dose-response curve values for KTC1 subpopulations treated with BRAF, MEK, and ERK1 inhibitors, relative to no inhibitor treatment of the same subpopulation or relative to KTC1-DMSO cells at the same inhibitor concentration

			BRAFi Dose-Response		MEKi Dose-Response		ERKi Dose-Response	
	log(pmol/L)	µM	Relative to no inhibitor	Relative to KTC1-DMSO	Relative to no inhibitor	Relative to KTC1-DMSO	Relative to no inhibitor	Relative to KTC1-DMSO
KTC1-DMSO	2.0	0.000	0.947	1.000	0.994	1.000	1.006	1.000
	3.0	0.001	0.885	1.000	0.971	1.000	0.974	1.000
	4.0	0.010	0.807	1.000	0.921	1.000	0.905	1.000
	5.0	0.100	0.720	1.000	0.848	1.000	0.798	1.000
	5.7	0.501	0.630	1.000	0.753	1.000	0.662	1.000
	6.0	1.000	0.555	1.000	0.648	1.000	0.511	1.000
	6.5	3.162	0.488	1.000	0.553	1.000	0.367	1.000
	6.7	5.012	0.403	1.000	0.450	1.000	0.252	1.000
	7.0	10.000	0.304	1.000	0.352	1.000	0.166	1.000
	7.7	50.119	0.202	1.000	0.254	1.000	0.103	1.000
	8.0	100.000	0.112	1.000	0.167	1.000	0.063	1.000
KTC1-VEM1	2.0	0.000	0.965	1.019	1.009	1.015	1.001	0.996
	3.0	0.001	0.904	1.021	1.021	1.051	0.952	0.977
	4.0	0.010	0.820	1.016	1.030	1.117	0.878	0.971
	5.0	0.100	0.713	0.991	1.011	1.192	0.773	0.969
	5.7	0.501	0.590	0.938	0.973	1.293	0.644	0.973
	6.0	1.000	0.470	0.847	0.917	1.414	0.502	0.984
	6.5	3.162	0.362	0.741	0.824	1.490	0.372	1.016
	6.7	5.012	0.258	0.641	0.678	1.508	0.257	1.021
	7.0	10.000	0.168	0.551	0.513	1.458	0.156	0.940
	7.7	50.119	0.094	0.466	0.350	1.381	0.075	0.731
	8.0	100.000	0.045	0.397	0.211	1.260	0.030	0.473
KTC1-VEM2	2.0	0.000	0.972	1.026	0.994	1.000	1.021	1.016
	3.0	0.001	0.952	1.075	0.993	1.023	1.001	1.028
	4.0	0.010	0.928	1.151	0.993	1.078	0.958	1.059
	5.0	0.100	0.901	1.253	0.981	1.156	0.875	1.097
	5.7	0.501	0.868	1.378	0.962	1.279	0.754	1.139
	6.0	1.000	0.818	1.475	0.929	1.433	0.607	1.189
	6.5	3.162	0.737	1.510	0.852	1.539	0.452	1.233
	6.7	5.012	0.603	1.498	0.711	1.581	0.311	1.235
	7.0	10.000	0.448	1.473	0.534	1.517	0.194	1.170
	7.7	50.119	0.287	1.422	0.358	1.410	0.100	0.976
	8.0	100.000	0.149	1.327	0.203	1.213	0.047	0.740

Supplementary Table S4: Primary and secondary antibodies used in this study

Primary Antibodies

Target	Supplier	Catalog #	Species	Dilution	Diluent
β-actin	Cell Signaling	4970	Mouse	1:1000	TBS w th 0.1% Tween 20, 5% BSA
ERK1/2	Cell Signaling	4695	Rabbit	1:1000	TBS with 0.1% Tween 20, 5% BSA
ERK1/2 (Thr202/Tyr204)	Cell Signaling	4370	Rabbit	1:1000	TBS with 0.1% Tween 20, 5% BSA
AKT	Cell Signaling	4691	Rabbit	1:1000	TBS with 0.1% Tween 20, 5% BSA
AKT (Ser473)	Cell Signaling	4060	Rabbit	1:2000	TBS with 0.1% Tween 20, 5% BSA
MET	Cell Signaling	8198	Rabbit	1:1000	TBS with 0.1% Tween 20, 5% BSA
MET (Tyr1349)	Cell Signaling	3133	Rabbit	1:1000	TBS with 0.1% Tween 20, 5% BSA
EGFR	Cell Signaling	2232	Rabbit	1:1000	TBS with 0.1% Tween 20, 5% BSA
EGFR (Tyr1068)	Cell Signaling	3777	Rabbit	1:1000	TBS with 0.1% Tween 20, 5% BSA
HER3	Cell Signaling	12708	Rabbit	1:1000	TBS with 0.1% Tween 20, 5% milk
HER3 (Tyr1289)	Cell Signaling	4791	Rabbit	1:1000	TBS with 0.1% Tween 20, 5% BSA
KRAS (G12D mutant)	Cell Signaling	14429	Rabbit	1:1000	TBS with 0.1% Tween 20, 5% milk

Secondary Antibodies

Target	Supplier	Catalog #	Species	Dilution	Conjugate
Mouse IgG	LI-COR	9266807	Goat	1:15,000	IRDye 680RD
Rabbit IgG	LI-COR	9263221	Goat	1:15,000	IRDye 800CW
